# Distribution of immunoglobulin G antibody secretory cells in small intestine of Bactrian camels (*Camelus bactrianus*)

**DOI:** 10.1186/s12917-015-0538-y

**Published:** 2015-08-25

**Authors:** Wang-Dong Zhang, Wen-Hui Wang, Shuai Jia

**Affiliations:** College of Veterinary Medicine, Gansu Agricultural University, Lanzhou, Gansu China

**Keywords:** IgG ASCs, Small intestine of Bactrian camel, Distribution, Mucosal immunity

## Abstract

**Background:**

To explore the morphological evidence of immunoglobulin G (IgG) participating in intestinal mucosal immunity, 8 healthy adult Bactrian camels used. First, IgG was successfully isolated from their serum and rabbit antibody against Bactrian camels IgG was prepared. The IgG antibody secretory cells (ASCs) in small intestine were particularly observed through immumohistochemical staining, then after were analyzed by statistical methods.

**Results:**

The results showed that the IgG ASCs were scattered in the lamina propria (LP) and some of them aggregated around of the intestinal glands. The IgG ASCs density was the highest from middle segment of duodenum to middle segment of jejunum, and then in ended segment of jejunum and initial segment of ileum, the lowest was in initial segment of duodenum, in middle and ended segment of ileum.

**Conclusions:**

It was demonstrated that the IgG ASCs mainly scattered in the effector sites of the mucosal immunity, though the density of IgG ASCs was different in different segment of small intestine. Moreover, this scatted distribution characteristic would provide a morphology basis for research whether IgG form a full-protection and immune surveillance in mucosal immunity homeostasis of integral intestine.

## Background

A conventional IgG is composed of two H and two L chains. It is the most abundant protein of plasma. They are synthesized and secreted by ASCs in spleen and lymph nodes, and their half-life is about 20 ~ 23 days.

The IgG subclasses and molecular characteristics are different in different animal species. Camelids (such as *Bactrian camel, Camelus dromedarius* and *Lama glama*) IgG differ from all other known antibodies and contradict all common theories on antibody diversity [1]. It is well established that camelids IgG have three subclasses (IgG1, IgG2 and IgG3) [2, 3], of which IgG1 composed of two H and two L chains is the conventional antibody, up to 25 % of circulating IgG. According to the different light chains (kappa and lambda), IgG1 was divided into IgG1a and IgG1b isotypes [4]. IgG2 and IgG3 composed of homodimeric H chain devoid of L chains are referred to as H chain Abs (HCAbs). They lack CH1 region, up to 75 % of circulating IgG. The IgG2, with long hinge, was divided into IgG2a, IgG2b and IgG2c isotypes in Lama and IgG2a and IgG2c isotypes in camels. IgG3 has short hinge [2, 5, 6].

At present, applied research of HCAbs has become the focus of attention. Because their antigen-binding domain consists of a single variable domain (referred to as VHH), which have smaller size [3], high level and stable expression in many vectors (such as *Escherichia coli* [7], *Saccharomyces cerevisiae* [8], tobacco plants [9] and Lactobacilli [10]), better tissue penetration, enlarge the antigen binding repertoire [11] and low immunogenicity. It is a useful tool for treating some diseases [12] (such as anti-diphtheria toxin [13], anti-α-cobratoxin [14]).

However, the research about the immunity system of camels are limited. Mucosal immunity plays an important role in the whole immunity system. But the function of the IgG in camel mucosal immunity has not been reported at present. Bactrian camel is an important livestock of economic characteristics in northwest of China. On the basis of our associated research with Bactrian camel mucosal immunity [15–19], the distribution of IgG ASCs in different sites of small intestine and the locating relationship of the distribution of IgG ASCs and MALT in small intestine of Bactrian camels (*Camelus bactrianus*) was preliminarily reported in this paper. We hope that it will provide the necessary support of the immunomorphology for further study whether HCAbs could participate in intestinal mucosal immunity or not.

## Methods

### Ethics statement

All experimental procedures were approved by the welfare authority of Minqin County of Gansu Province.

### Experimental animals and serum preparation

Eight clinically normal Alashan Bactrian camels (half male and female, 3–5 years) were anaesthetised with sodium pentobarbital and exsanguinated. The blood samples were collected from the jugular, and serum was isolated and preserved at −20 °C refrigerator for use.

Two New Zealand white male rabbits aged 8 weeks were bought from Experimental Animal Center of Lan Zhou Veterinary Research Institute of the Chinese Academy of Agricultural Sciences (CAAS).

### IgG extraction and purification

A stock solution of saturated ammonium sulfate (SAS) was prepared and stored at room temperature (approximately 25 °C). The 100 % SAS was slowly added into the experimental sample (serum to normal saline was 1:1(v/v)) and was gently stirred to mix well, then resulted in reaction mixtures of 20 % SAS. The reaction mixture was set aside at 4 °C for 2 h and then centrifuged to pack the precipitated protein. Then 100 % SAS was added into the supernatant fluid continually, and resulted in reaction mixtures of 25, 30, 35, 40, 45, 50, 55, and 60 % SAS and repeated above steps, centrifuged to pack the precipitated protein and stored for later analysis [20–22]. According to the concentrations, molecule weight and structural characteristic of Bactrian camels IgG, the accurate percentages of ammonium sulfate precipitating IgG was determined by sodium dodecyl sulfate-polyacrylamide gel electrophoresis (SDS-PAGE). Finally, the crude extraction was further purified by sephadex G-200 column and DEAE-52 ion exchange column in sequence. The protein purifications was identified by the SDS-PAGE.

### Rabbit antibody against Bactrian camels IgG preparation

Rabbit antibody against Bactrian camels IgG was prepared by hybrid immune stimulating and the antibody titer was determined by immuno-double diffusion [23].

### Microsection

The abdomen of every Bactrian camel was incised and the whole small intestine from pylorus of abomasal to ileocecal aperture was taken out. Histological samples of the duodenum were taken in order of initial segment, middle segment and distal segment, and they were similar to jejunum and ileum. All samples were fixed in 4 % neutral paraformaldehyde solution for more than 15 days. A paraffin sections were obtained by routine method and stained with SABC-immunohistochemistry.

Primary antibodies: rabbit polyclonal antibodies against Bactrian camels IgG were from our laboratory (Veterinary pathology laboratory of college of veterinary medicine, Gansu Agricultural University, China). The best working concentration of the primary antibodies was 1:1200.

Second antibodies: SABC goat anti-rabbit polyclonal antibodies immunohistochemical kit (Lot No.07H3OCJ, Boster, Wuhan, Hubei, China).

### Light microscopy

The distribution location and characteristics and density of IgG ASCs in each segment were carefully observed under the microscope. 30 sections were observed and photomicrographed using Olympus DP-71 microscopy system in each segment.

### Statistical analysis

Five sections were randomly selected for each. 10 microscopic fields were randomly selected in every section, and were observed and photomicrographed. The number of positive ASCs in every microscopic field was counted and the destiny was calculated (Image-Pro Plus 6.0). Data analysis was performed using the Duncan’s new multiple range method using IBM SPSS 17.0 statistics software for Windows software (IBM software, Chicago, USA). Differences were 5 % significant level.

## Results

### IgG extraction and purification

**Fig. 1:**
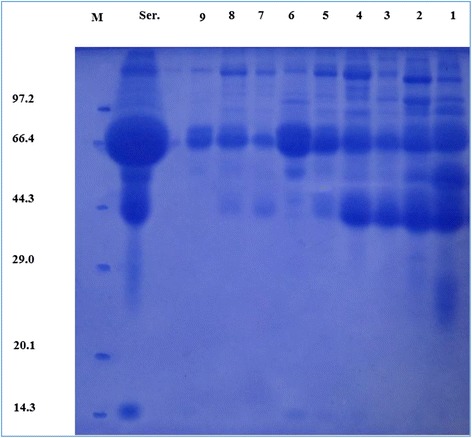
SDS-PAGE results. 1–9 lanes are crude extraction obtained using ammonium sulfate solution in saturation 20 %, 25 %, 30 %, 35 %, 40 %, 45 %, 50 %, 55 % and 60 % in sequence. Ser. lane is the serum samples. M lane is the proteins marker

**Fig. 2:**
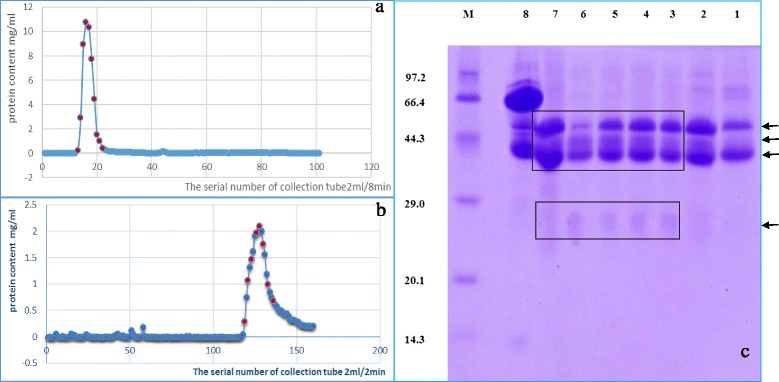
**a** sephadex G-200 column elution curves, there are only single peak from the 13th to the 22th collection tubes which were marked red dot. **b** DEAE-52 ion exchange column elution curves, the gradient elution with phosphate buffer (0.01–0.3 mol/L, pH 8.0), there are only single peak. **c** SDS-PAGE results: lanes 1–7 are respectively the 136th, 133th, 130th, 128th, 126th, 123th, 121th collection tube which were marked red dot in (**b**). the 8th lane is the concentrations of 13–22 collection tubes in (**a**). *M* lane is the protein marker. The molecular weight of IgG1, IgG2 and IgG3 was 164.6, 94.7 and 89.4 kDa respectively

SDS-PAGE results showed that the IgG were mainly was found lying in saturation with ammonium sulfate in the range of 30–35 % (Fig. 1). The further purified results showed that sephadex G-200 column and DEAE-52 ion exchange column elution curves were both single peak (Fig. 2a-b), and the purified IgG were determined on 3–7 lanes of SDS-PAGE (Fig. 2-c). By the calculation, the molecular weight of IgG1, IgG2 and IgG3 was 164.6, 94.7 and 89.4 kDa respectively.

### Results analysis of Bactrian camels IgG purification

Salting-out results detected by SDS-PAGE showed that the content and components of proteins in precipitates gradually decreased as the increase of the ammonium sulfate saturation. Ser. lane (Fig. 1) showed that the most abundant protein was found at the position of about 66.4 kDa and the second was at the position of about 44.3 kDa. It was reported that the concentration of albumin was the highest. The concentration of γ-globulin was the second [24, 25]. So the protein at the position of about 66.4 kDa should be albumin and at the position of about 44.3 kDa should be γ-globulin. Moreover, IgG content accounted for 75 % in γ-globulin, was the highest, followed by IgA, and IgM was the least. Zhang L. J. reported that the molecular weights of Bactrian camel IgA and IgM heavy chain were higher than IgG heavy chain [26]. So Bactrian camel IgG was at the position of about 44.3 kDa in the third and fourth lane (Fig. 1). We could conclude, that the saturation percentages of ammonium sulphate precipitating Bactrian camel IgG were 30–35 %. This saturation percentage was a little lower than the saturation of ammonium sulphate precipitating Bactrian camel IgG in colostrum which was 40 % reported by Hongbo et al. [27]. It was close to the saturation of ammonium sulphate precipitating human and mice IgG in blood which was 30 % [28]. Analyzing the reasons causing the differences, we found that the higher the concentration of total protein in the sample, the lower the saturation of ammonium sulphate precipitating target protein. So the concentration of sample proteins should be generally in the range of 25.0 ~ 30.0 g/L.

The results of further purification detected by SDS-PAGE gel electrophoresis (Fig. 2-c) showed that from the third line to the seventh line in each of which three protein bands appeared near the position of 44.3 kDa, which were consistent with the molecular weights of heavy chains of three subtypes Bactrian camel IgG. And there was also a protein band at the position of between 29.0 and 20.1 kDa, which was consistent with the molecular weight of light chain of Bactrian camel IgG1. This band was slightly stained, because in Bactrian camel IgG subsets only IgG1 had light chains and IgG2 and IgG3 were naturally lack of light chains. In 2011. Tillib reported that the molecular weight of the IgG1 was approximately 150 kDa and its heavy chain molecular weight was 52–55 kDa; the molecular weights of IgG2 and IgG3 were approximately 80 ~ 90 kDa and their heavy chains molecular weights were about 40 ~ 47 kDa. HCAbs accounted for 60 ~ 80 % in Bactrian camel IgG [3]. Based on above analysis, the further purified proteins were all Bactrian camel IgG. In the first lane and the second lane, besides two obvious protein bands appeared near the position of about 44.3 kDa, one protein band also appeared near the position of about 66.4 kDa. So we thought of this part of samples were not purified and abandoned.

The protein components of purifications eluted using Sephadex G-200 column in the eighth line (Fig. 2-c) compared with the fourth line (Fig. 1) greatly reduced and weren’t purified. It indicated that Sephadex G-200 column could only isolate different components whose molecular weights were great different. In general, pure target protein were quickly obtained by combining gel chromatography (such as Sephadex G-200 column) and ion-exchange column chromatography (such as DEAE-52 ion exchange column) during experiment. In this way, three IgG subsets (IgG1, IgG2 and IgG3) could be isolated and obtained in only one experiment, extraction volume was bigger, the processing steps were more simple, which compared with the method of the immunoaffinity chromatography (protein A, protein G and gel filtration) [2].

### Antibody titer

Rabbit anti-Bactrian camel IgG antibody titer detected by immuno-double diffusion was 1:512, which well met the sequent experiment request.

### The distribution characteristics of IgG ASCs in small intestine of Bactrian camels

**Fig. 3:**
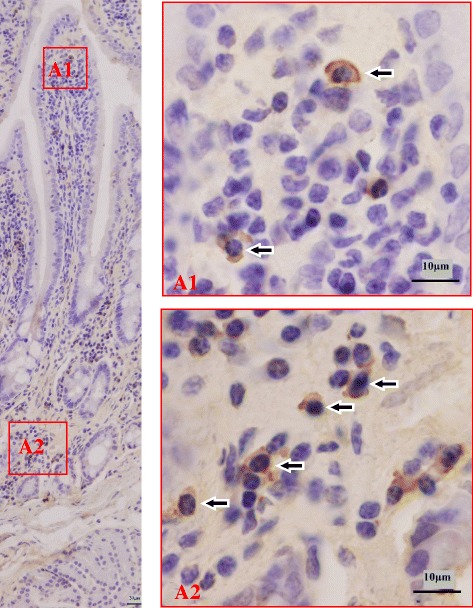
Distribution pattern of IgG ASCs in the duodenum. The left large picture presents an overview of a typical Bactrian camel duodenum structure with sublocalizations *A1-A2* in frames. Small pictures *A1-A2* illustrate representative views from the two sublocalizations. Cells labeled positively for IgG ASCs show brown staining (*arrowhead*)

**Fig. 4:**
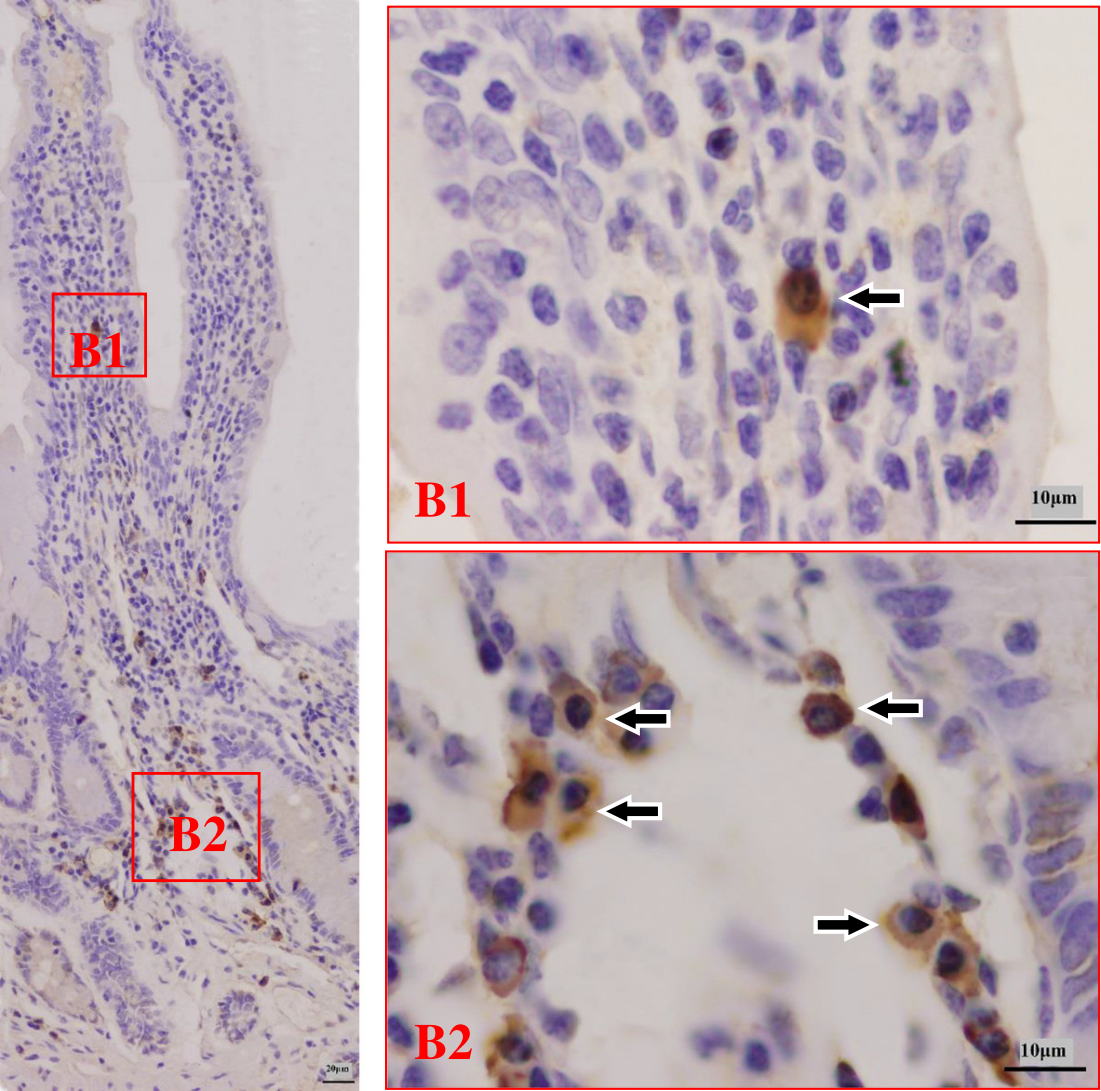
Distribution pattern of IgG ASCs in the jejunum. The left large picture presents an overview of a typical Bactrian camel jejunum structure with sublocalizations *B1-B2* in frames. Small pictures *B1-B2* illustrate representative views from the two sublocalizations. Cells labeled positively for IgG ASCs show brown staining (*arrowhead*)

**Fig. 5:**
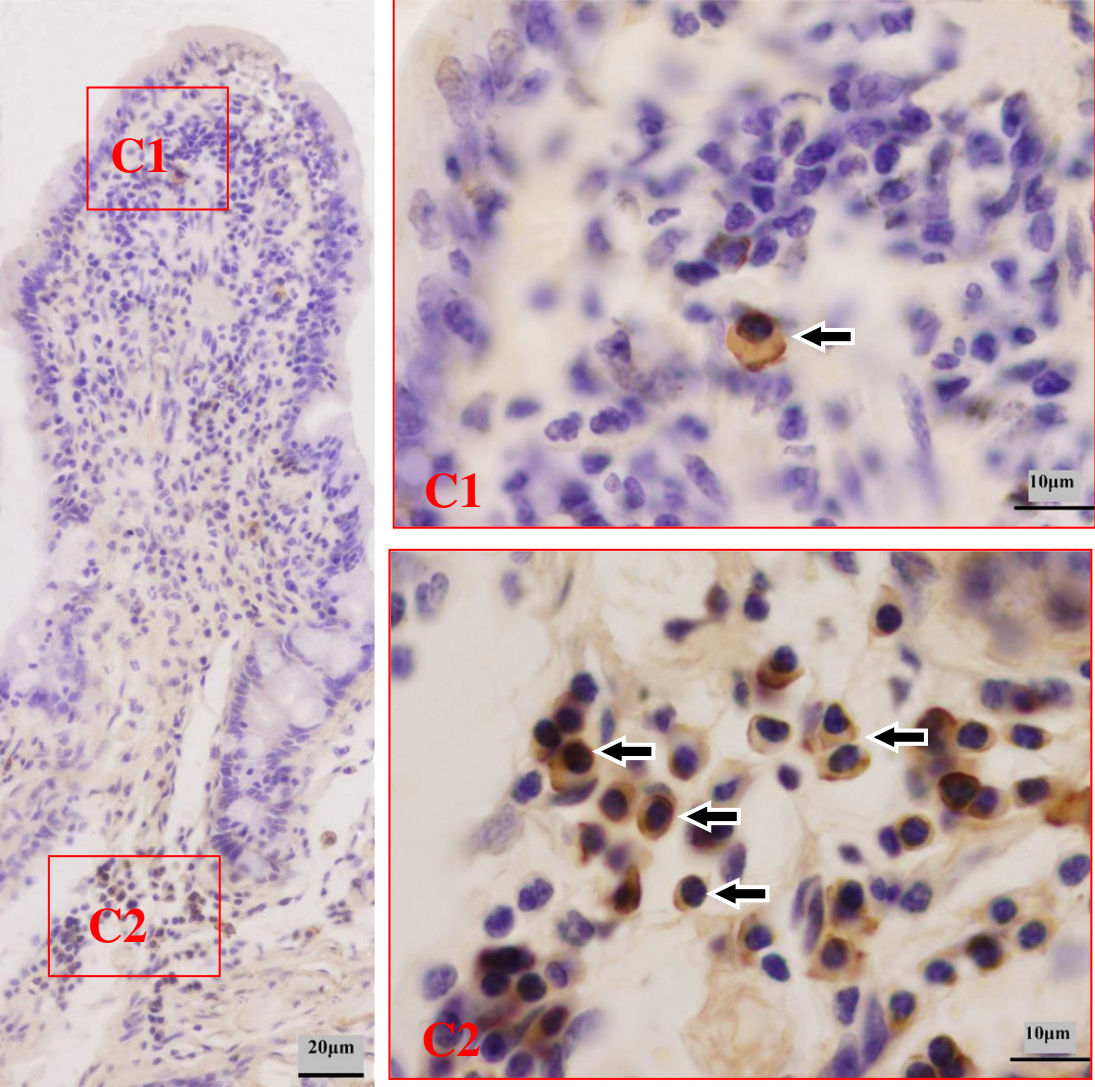
Distribution pattern of IgG ASCs in the ileum. The left large picture presents an overview of a typical Bactrian camel ileum structure with sublocalizations *C1-C2* in frames. Small pictures *C1-C2* illustrate representative views from the two sublocalizations. Cells labeled positively for IgG ASCs show brown staining (*arrowhead*)

The results of observation showed that the distributions of IgG ASCs in the duodenum, jejunum and ileum were basically similar, some of them were scattered in the LP and some of them aggregated around of the intestinal glands (Figs. 3, 4 and 5). Furthermore,and a few were sporadically scattered in the dome areas of the aggregated lymphoid nodules and diffuse lymphoid tissue. IgG ASCs were not observed in the aggregated lymphatic follicles, solitary lymphatic and interfollicular area.

### The distribution density of IgG ASCs in small intestine of Bactrian camels

**Fig. 6:**
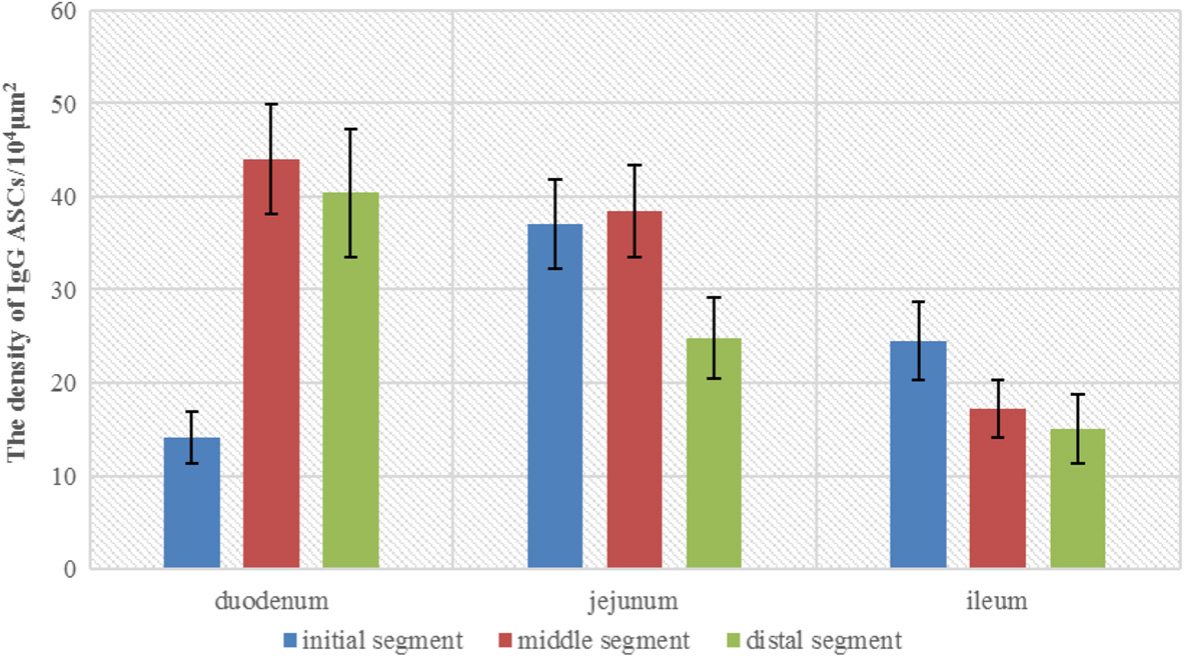
Bar graph of the IgG ASCs’ density. The density of IgG ASCs was in each segment of Bactrian camel small intestine (unit: /10^4^ μm^2^)

Analysis result showed that addition to initial segment of duodenum, the density of IgG ASCs was declined from middle segment of duodenum to distal ileum (Fig. 6). The density of IgG ASCs was highest in the middle segment of duodenum (44.00 ± 5.89), lowest in the initial segment of duodelum (14.11 ± 2.82). It was significant higher in middle (44.00 ± 5.89), distal (40.41 ± 6.86) segment of duodenum and in initial (37.05 ± 4.87), middle (38.47 ± 4.93) segment of jejunum than other segments (*P* < 0.05). But it was significant lower in initial segment of duodenum (14.11 ± 2.82) and in middle (17.16 ± 3.09), distal (15.03 ± 3.67) segment of ileum (*P* < 0.05) (Table 1).

**Table 1:** The distributed density of IgG ASCs in the small intestinal LP of Bactrian camels (Mean ± SD) unit:/10^4^ μm^2^

	Initial segment	Middle segment	Distal segment
Duodenum	14.11 ± 2.82^f^	44.00 ± 5.89^a^	40.41 ± 6.86^b^
Jejunum	37.05 ± 4.87^c^	38.47 ± 4.93^bc^	24.79 ± 4.38^d^
Ileum	24.49 ± 4.17^d^	17.16 ± 3.09^e^	15.03 ± 3.67^ef^

All data in table marked different lowercase letter differ significantly (*P* < 0.05)

## Discussion

The digestive tract mucosal immune system can be mainly divided into two parts mucosal immunity induction area and effector sites according to their function characteristic. The mucosal immunity induction area was mainly composed of aggregated lymphatic follicles and solitary lymphatic follicles. Our research results indicated that the IgG ASCs were scattered in the LP and some of them aggregated around of the intestinal glands. The IgG ASCs density was the highest from middle segment of duodenum to middle segment of jejunum, and then in distal segment of jejunum and initial segment of ileum, the lowest was in initial segment of duodenum, in middle and distal segment of ileum. However, the results of the research on the distribution of the Bactrian camel intestinal Peyer’s patches (PPs) showed that the PPs were mainly distributed in the Ileum and there were less in the duodenum and jejunum [17]. Moreover, this distribution characteristics were similar to those in human, rat, cow and sheep’s intestine [29]. The distribution trends of the PPs and the IgG ASCs in the intestine were exactly opposite each other. In other words, the PPs were mainly distributed in mucosal immunity induction area, but the IgG ASCs were mainly distributed in the effector sites. In addition, the results of the research on the distribution of the Bactrian camel SIgA ASCs showed that the distribution characteristics were similar to those of IgG ASCs [26]. Bactrian camel SIgA ASCs were mainly distributed in mucous membranes LP around intestinal gland, which were also belonged to the effector sites of mucosal immunity [26]. Moreover, this scatted distribution characteristic would be benefit for IgG to form a full-protection and immune surveillance in mucosal immunity homeostasis of integral intestine. At present, it was reported that the receptor FcγR (such as FcγR, FcγRIIor FcγRIII) was expressed on the surface of most mucosal immunity cells (such as macrophage, dendritic cell, NK cell, mast cell and granulocyte) [30]. And the function of these cells could be regulated by the reaction between IgG and its receptor. Moreover, some studies suggested that in small intestinal mucosal immunity, IgG provided the second line of defense that controls microbial dissemination by eliciting a robust inflammatory reaction [31–33].

SIgA played a crucial role in mucosal immunity, of which important reason is that SIgA could form a protective layer in the lumen by transcytosis of PIgR [34–36]. Similarly, recent studies demonstrated that the neonatal Fc receptor for IgG (FcRn) which was a kind of receptor of IgG were also expressed in the intestinal epithelial cells in newborns [37, 38]. FcRn could be combined with the Fc portion of IgG at lower than physiological pH (<6.5) and released at a physiological pH (7.4). Thus IgG could be bidirectionally transported between lumen and mucosa. And in this paper, the study of the distribution of IgG ASCs in intestinal mucosa laid the foundation for further studying Bactrian camel’s IgG, especially whether the unique HCAb participating in Bactrian camel’s intestinal mucosal immune response through FcRn’s bidirectional.

## Conclusions

This study demonstrated that Bactrian camel IgG ASCs were mainly diffusely distributed in non-PPs area in mucous membranes LP of small intestine. This area was mucosal immunity induction area in intestine. This study provided a morphology basis for research whether IgG form a full-protection and immune surveillance in mucosal immunity homeostasis of integral intestine.

## Abbreviations

IgG: Immunoglobulin G
ASCs: Antibody secretory cells
LP: Lamina propria
HCAbs: H chain Abs
SAS: Saturated ammonium sulfate
SDS-PAGE: Sodium dodecyl sulfate-polyacrylamide gel electrophoresis
PPs: Peyer’s patches
FcRn: Neonatal Fc receptor for IgG
